# Towards a Tissue-Engineered Contractile Fontan-Conduit: The Fate of Cardiac Myocytes in the Subpulmonary Circulation

**DOI:** 10.1371/journal.pone.0166963

**Published:** 2016-11-22

**Authors:** Daniel Biermann, Alexandra Eder, Florian Arndt, Hatim Seoudy, Hermann Reichenspurner, Thomas Mir, Arlindo Riso, Rainer Kozlik-Feldmann, Kersten Peldschus, Michael G. Kaul, Tillman Schuler, Susanne Krasemann, Arne Hansen, Thomas Eschenhagen, Jörg S. Sachweh

**Affiliations:** 1 Cardiac Surgery for Congenital Heart Disease, University Heart Center Hamburg, University Medical Center Hamburg-Eppendorf, Hamburg, Germany; 2 Department for Paediatric Cardiology, University Heart Center Hamburg, University Medical Center Hamburg-Eppendorf, Hamburg, Germany; 3 Department for Cardiovascular Surgery, University Heart Center Hamburg, University Medical Center Hamburg-Eppendorf, Hamburg, Germany; 4 Department of Experimental Pharmacology and Toxicology, University Medical Center Hamburg-Eppendorf, Hamburg, Germany; 5 Department of Diagnostic and Interventional Radiology, University Medical Center Hamburg-Eppendorf, Hamburg, Germany; 6 Institute of Neuropathology, University Medical Center Hamburg-Eppendorf, Hamburg, Germany; 7 DZHK (German Center for Cardiovascular Research), partner site Hamburg/Kiel/Lübeck, Hamburg, Germany; University of Torino, ITALY

## Abstract

The long-term outcome of patients with single ventricles improved over time, but remains poor compared to other congenital heart lesions with biventricular circulation. Main cause for this unfavourable outcome is the unphysiological hemodynamic of the Fontan circulation, such as subnormal systemic cardiac output and increased systemic-venous pressure. To overcome this limitation, we are developing the concept of a contractile extracardiac Fontan-tunnel. In this study, we evaluated the survival and structural development of a tissue-engineered conduit under in vivo conditions. Engineered heart tissue was generated from ventricular heart cells of neonatal Wistar rats, fibrinogen and thrombin. Engineered heart tissues started beating around day 8 in vitro and remained contractile in vivo throughout the experiment. After culture for 14 days constructs were implanted around the right superior vena cava of Wistar rats (n = 12). Animals were euthanized after 7, 14, 28 and 56 days postoperatively. Hematoxylin and eosin staining showed cardiomyocytes arranged in thick bundles within the engineered heart tissue-conduit. Immunostaining of sarcomeric actin, alpha-actin and connexin 43 revealed a well -developed cardiac myocyte structure. Magnetic resonance imaging (d14, n = 3) revealed no constriction or stenosis of the superior vena cava by the constructs. Engineered heart tissues survive and contract for extended periods after implantation around the superior vena cava of rats. Generation of larger constructs is warranted to evaluate functional benefits of a contractile Fontan-conduit.

## Introduction

**Fig 1:**
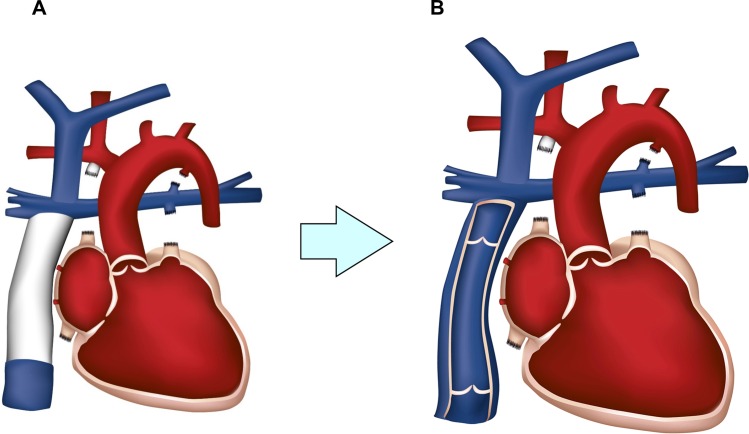
The concept of a subpulmonary „neo-ventricle”from engineered heart tissue. A: Extracardiac tunnel for the treatment of children with univentricular hearts. Commonly, a non-contractile GoreTex-conduit is used to bypass blood from the inferior caval vein to the right pulmonary artery. B: Our group aims for a contractile, valved conduit made from engineered heart tissue (EHT) to propel blood actively through the lungs and avoid endorgan damage in the long-term.

The Fontan principle is the only surgical treatment for patients with single ventricle anatomy aiming at separation of systemic and pulmonary circulation[1]. Since Francis Fontan published his technique in 1971 many surgical modifications have been applied[2, 3]. All these modifications were based on the experience that Fontan patients have a relevant morbidity and mortality in comparison to patients after biventricular repair[4–6]. Even in the modern era freedom from death or transplantation in a recent study was 87%, 83% and 70% after 15, 20 and 25 years, respectively[7]. The key problem is, named by de Leval, the “Fontan paradox”, i.e. a caval hypertension and pulmonary hypotension[8]. Due to the absent subpulmonary ventricle there is no significant driving force and systemic venous pressure is immediately related to the pulmonary artery pressure. Increased systemic venous pressure leads to the typical endorgan sequelae of hepatic cirrhosis, protein losing enteropathy, ascites and plastic bronchitis. To overcome this, creation of a valved subpulmonary ventricle with the principles of tissue engineering may be a valuable option. Our group aims at a valved „neo-ventricle”from engineered heart tissue (EHT) as an extracardiac pumping chamber (Fig 1). Towards this goal, as a first step we histologically evaluated the survival, sarcomeric integrity and vascularization of cardiomyocytes after implantation around the superior caval vein in a rat model.

## Materials and Methods

All procedures were approved by the local animal protection authority (BGV of the Freie und Hansestadt Hamburg: #101/13) and conformed to the *Guide for the Care and Use of Laboratory Animals* (NIH publication 86–23, revised 1996).

### Cell Harvest, Culture and Generation of Contractile Tissue Grafts

We generated engineered heart tissues from freshly isolated neonatal (day 0–3) rat heart cells. Single EHT-units were generated and cultured between flexible silicone posts in 24-well format as described previously in detail[9]. In short, fresh ventricular heart cells were mixed with fibrinogen, thrombin and medium. Subsequently, the mastermix (100 μl, containing 4.1x10^5^ cells) was casted in 12 x 3 x 3 mm molds. EHTs were implanted after day 14, prior to implantation EHTs were incubated with DAPI (1 μg/ml, Molecular Probes, Waltham, USA).

### Implantation and Follow-Up

Deeply anesthetized (3,5% sevoflurane, Baxter, Unterschleißheim, Germany) male Wistar rats (n = 12, 300–350 g, Charles River, Sulzfeld, Germany) were placed on a heated operation table (37.5°C). The chest was opened via right thoracotomy in the 3^rd^ intercostal space and the ribs were spread with a small animal retractor. Subsequently, the SVC was dissected. In each animal two EHT strips (6–8 mm length, 1 mm diameter) were wrapped around the SVC with approximately 1–2 mm distance to the right atrium. The ends were sutured using a single stitch 8–0 nylon (Prolene, Ethicon, Germany). Buprenorphine hydrochloride (0.1 mg/kg, intramuscular injection) was applied during surgery and for additional three days. For immunosuppression, cyclosporine A (5 mg/kg) and methylprednisolone (2 mg/kg) were administered daily by subcutaneous injection with a 27 G canula (BD, Franklin Lakes, USA). Animals (n = 3 each) were sacrificed after 7, 14, 28 and 56 days, respectively. In addition, three animals for MRI analysis (d14) underwent sham operation without implantation of a tissue graft.

### Morphological Analysis

“Neo-ventricles” were fixed in formaldehyde, embedded in paraffin and cross sections were taken for staining procedures. Since EHTs were labeled with DAPI prior to implantation, sections were dewaxed and mounted with Fluoromount-G (SouthernBiotech, Birmingham, USA) to identify implanted EHT donor cells in the vicinity of the vessel. Sarcomeric actin was labeled with an antibody (1:600; A7811, Sigma, St. Louis, USA) according to standard procedures with diaminobenzidine on deparaffinized sections, pretreated with protease for antigen retrieval for 8 min and evaluated with light microscopy using a Leica digital microscope (DMD108; Leica, Wetzlar, Germany). Tissue integrity of implanted EHTs around vessels was determined using immunofluorescence stainings. Heat mediated antigen retrieval was performed for 30 min at 96°C in 10 mM citrate buffer pH 6.0. Subsequently, sections were permeabilized with 0.2% Triton X-100 (Roche, Basel, Switzerland) in TBS. Tissues were blocked in Pierce Protein-Free T20 blocking buffer (Thermo Scientific, Waltham, USA) and treated with 1% Sudan Black to reduce autofluorescence. Antibodies directed against CD31 (1:100; SZ31, Dianova, Hamburg, Germany), alpha-actin (1:100; M0874, Dako, Hamburg, Germany) and connexin 43 (1:100; 610061, BD, Franklin Lakes, USA) were incubated overnight at 4°C and for detection the appropriate fluorescently labeled secondary antibodies were used. Data were acquired with a Leica TCS SP5 confocal microscope with 63x objective using Leica Application Suite software (LAS-AF-lite).

### Magnetic Resonance Imaging (MRI)

To evaluate possible constriction of the SVC by EHTs in vivo MRI was performed. Animals were anesthetized with 2% isoflurane. Coronal cine gradient echo images with a temporal resolution of 6 ms were acquired in a segment of the SVC caudal to the "neo-ventricle" on a 7T MRI (Bruker ClinScan 70/30 USR, Ettlingen, Germany) using respiratory gating and pulse oximetry triggering (Model 1025T Sa Instruments, Stony Brook, USA).

### Statement of Responsibility

The authors had full access to the data and take full responsibility for its integrity. All authors have read and agree to the manuscript as written.

## Results

### Generation of a “Neo-Ventricle” from Engineered Heart Tissue and Graft Survival

**Fig 2:**
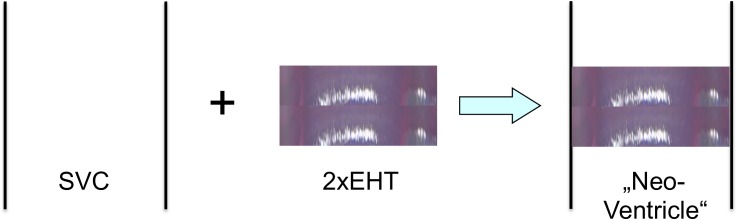
Generation of a simple contractile conduit from EHT. The illustration shows the construction of a simple „neo-ventricle”out of engineered heart tissue. Two lamellar EHTs were wrapped around the right SVC of adult Wistar rats. The ends were sutured using a single stitch of nylon suture.

EHTs started beating around day 8 and were implanted around the SVC of Wistar rats after 14 days in vitro cultivation (Fig 2, n = 12). Animal weight was 329±14 g on the day of implantation. No major bleeding was documented and all animals survived circumferential implantation of the grafts. Most conduits remained visibly contractile for the rest of the experiment (d7: 3/3, d14: 2/3, d28: 3/3, d56: 2/3). The contractility was observed microscopically (20 x magnification) after explantation of the conduit.

### Histology and Vascularization

**Fig 3:**
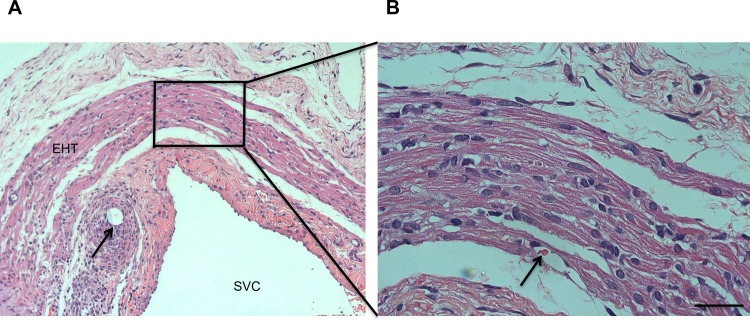
Hematoxylin and eosin (HE) staining of “neo-ventricle” 56 days after implantation around the superior caval vein (SVC) of rats. A: Engineered heart tissue organized around the SVC. Thick bundles of cross-striated cardiomycytes are shown above the smooth muscle of the native vessel wall. The arrow indicates a suture with surrounding lymphocytic infiltration. B: Magnification of detail in left panel. Cross-striation can be seen in the highly organized cardiomyocytes within the contractile conduit. The arrow indicates red blood cells in a capillary. Scale bar: 50 μm.

**Fig 4:**
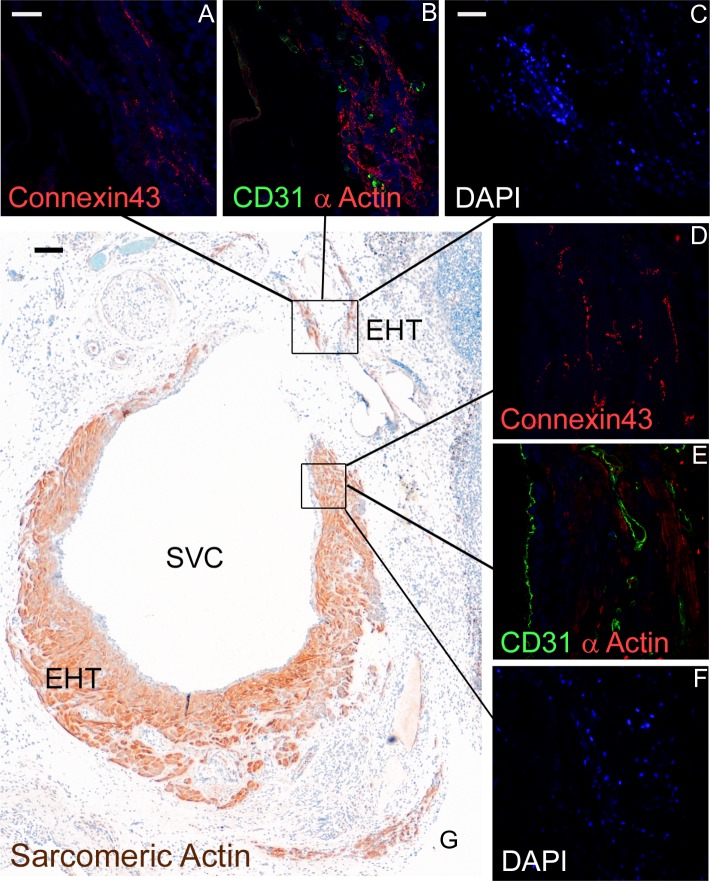
Cardiac morphology of EHT-conduit 28 days after grafting. A+D: Connexin 43 (red), an important gap junction protein indicating functional syncytium of the grafted cells is shown. Scale bar: 20 μm. B+E: CD31 staining (green) revealed dense vascularization. Vessels displayed a small diameter compatible with capillaries. Alpha-actin staining (red) showed dense bundles of cross-striated cardiomyocytes around the SVC. C+F: DAPI (blue) labeling of cells before implantation allowed for clear identification of grafted cells after harvest of the specimen. Scale bar: 50 μm. G: Overview of depicted (A-F) sarcomeric details showing the circumferential alignment of the „neo-ventricle“. Scale bar: 100 μm.

**Fig 5:**
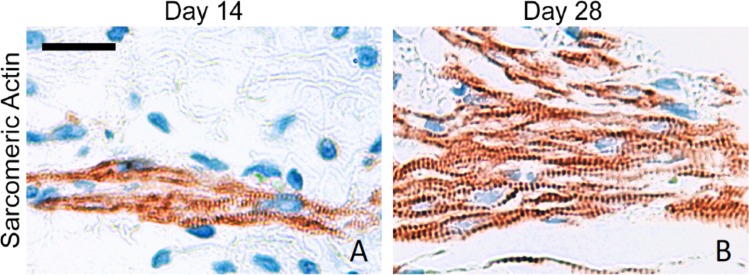
Sarcomeric integrity of implanted EHTs after 14 and 28 days in vivo. A+B: Cross-striation of cardiomyocytes in DAPI-positive areas is shown. Scale bar: 20 μm.

Tissue grafts could clearly be identified at all time points after implantation. The conduit showed an interconnected network of highly differentiated cardiomyocytes and rich vascularization (Fig 3A). Red blood cells (Fig 3B) were detected in the capillaries as a proof of connection to the native vasculature. Moreover, staining of sarcomeric actin demonstrated circumferential alignment around the SVC (Fig 4G) after 28 days. Abundant Connexin 43 (Fig 4A and 4D, red) as an indicator for a functional and electrical syncytium was observed in all “neo-ventricles” that were contractile at the end of the study. CD31 (Fig 4B and 4E, green) staining was used to show dense vascularization. DAPI (Fig 4F and 4C, blue) labeling showed substantial survival of cells within the contractile conduit and allowed clear identification of grafted cells after harvest of the specimen. Sarcomeric integrity with plenty of cross striated cells from a DAPI-positive area was shown in sarcomeric actin stainings (Fig 5).

### Magnetic Resonance Imaging

**Fig 6:**
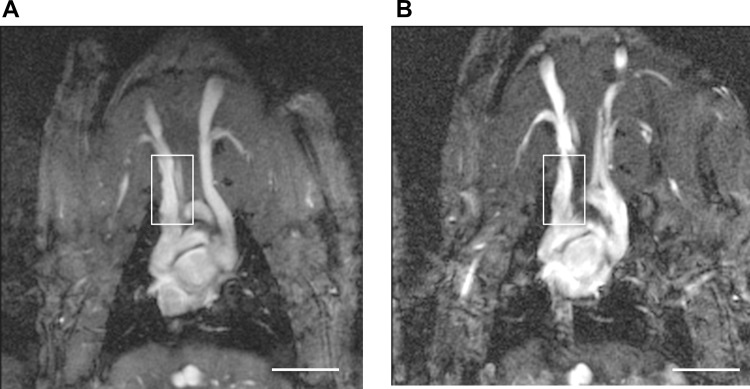
Cine MRI 14 days after implantation of EHTs around the SVC of rats. A: Approximately 1 mm above the right atrium two stripes of EHT were implanted around the SVC (box). No stenosis or constriction of the vein could be detected in the treatment group. B: The box shows the SVC of a Sham operated animal 14 days after the procedure. Scale bars: 1 cm.

In MRI the area of implantation could be clearly identified and no stenosis or compression by the construct was seen (Fig 6). Consequently, average vessel diameter was not reduced in the treatment group (control 1.4±0.11 mm vs. treatment group 1.46±0.12 mm, n.s.).

## Discussion

In Fontan patients, systemic venous hypertension is responsible for endorgan failure, like protein losing enteropathy or hepatic fibrosis, leading to death or heart transplantation[10, 11]. As heart transplantation is a limited option, several technologies, including mechanically assisted Fontan circulation and cardiomyoplasty procedures have been attempted[12–14]. As an alternative to aforementioned approaches the field of cardiac tissue engineering is emerging[15–17]. Tissue engineered cardiomyoplasty was introduced by several groups for the treatment of heart failure[18, 19]. Besides the treatment of acquired heart disease the field was also driven by the wish to cure congenital malformations that present with hypoplastic ventricular morphology[20]. This approach implies structural an electrical integration into the recipient myocardium.

Our group developed a concept to replace the missing right ventricle in univentricular circulation by an extracardiac subpulmonary “neo-ventricle” from EHT. Our study provides first evidence for the suitability of EHT as a transplantable heart muscle around the superior vena cava. The concept was tested in healthy Wistar rats in order to mimic a setting of venous preload for the utilized ventricular myocytes. Animal survival was 100% after lateral thoracotomy and implantation of EHTs around the SVC. Histology revealed sarcomeric integrity, a matured phenotype and adequate vascularization of implanted cardiomyocytes (up to 56 days). There was no electrical coupling of the implanted constructs to the native myocardium. Our study was not designed to assess functional performance or therapeutic effects of the rudimentary “neo-ventricle”. This question will be answered after testing these constructs as an extracardiac tunnel in larger animals.

Adequate vascularization of EHTs after implantation has been previously shown by our group in rat models of myocardial infarction[19]. Furthermore, an extensive, though primitive vascular network spontaneously develops in EHTs after culture in vitro[21]. EHTs were never tested under the investigated conditions of the venous system (laminar flow) with presumably altered angiogenic factors compared to the arterial system. In our setting EHTs were strongly vascularized. Functional vascularization enabling sufficient metabolite and oxygen supply is of prime importance to render survival of larger constructs in the future possible.

Confocal laser scanning microscopy showed a matured phenotype of cardiomyocytes in EHTs even under conditions of reduced preload around a great vein. Cross-striation and gap junction proteins were amply shown in the time course of the experiment. In addition, we noted that EHTs remained contractile for a considerable time after implantation and documented negligible graft failure. To exclude stenosis of the SVC after implantation we performed MRI analysis, which did not show any constriction of the vein.

Our study presents a novel concept of contractile elements implanted around a great vein in order to develop a pulsatile Fontan-conduit, which can actively propel blood through the lung. Future work will have to specifically work out the structural and functional requirements of such a pulsatile conduit. The final conduit will need to meet the dimensions of a growing child and must be from a autologous cell source (e.g. induced pluripotent stem cells) to avoid immunosupression. Furthermore, it must encompass tissue-engineered valves to direct the flow to the pulmonary arteries and avoid rising venous pressures. In the development of such a subpulmonary “neo-ventricle” the groundbreaking experiments of de Leval showing the energy losses at cavities, corners and stenoses must be considered. It was shown that the right atrium did not perform as an efficient pump in a valveless atriopulmonary connection. Consequentely, diastolic and systolic function of the construct must be followed to allow for forward flow towards the pulmonary circulation. In this context, computational fluid dynamics will have major impact on designing a bioartificial “neo-ventricle” at the bench. Tang and colleagues recently showed the impact of geometry on energy dissipation in patients undergoing total cavopulmonary connection[22]. Another question that needs to be addressed is the chronotropic control of the construct to achieve an adequate output to the pulmonary vasculature. The proof-of-concept for this approach must be provided from using a large animal model before translation into the clinical setting.
